# Preoperative preparation of eye with chlorhexidine solution significantly reduces bacterial load prior to 23-gauge vitrectomy in Swedish health care

**DOI:** 10.1186/s12886-018-0844-9

**Published:** 2018-07-11

**Authors:** Nasser J. Gili, Torbjörn Noren, Eva Törnquist, Sven Crafoord, Anders Bäckman

**Affiliations:** 1 Department of Ophthalmology, Örebro University Hospital, SE-701 85 Örebro, Sweden; 2 Faculty of Medicine and Health, Örebro University, Örebro, Sweden; 3 Department of Laboratory Medicine, Örebro University Hospital, Örebro, Sweden; 4 Department of Clinical Research Laboratory, Örebro University Hospital, Örebro, Sweden

**Keywords:** Conjunctival colonization, chlorhexidine solution, Endophthalmitis, Microorganisms, Small-gauge vitrectomy

## Abstract

**Background:**

Bacteria in the conjunctiva present a potential risk of vitreous cavity infection during 23-gauge pars plana vitrectomy (PPV). Current preoperative procedures used in Sweden include irrigation with chlorhexidine solution (CHX) 0.05% only and no iodine solutions. We evaluated the bacterial diversity and load before and after this single antibacterial measure.

**Methods:**

In a prospective, consecutive cohort we investigated bacterial growth in samples from 40 eyes in 39 consecutive individuals subjected to vitrectomy. A conjunctival specimen was collected from each preoperative patient before and after irrigating of eye with CHX, 0.05% solution. Iodine was not used during any part of the surgery. One drop of chloramphenicol was administered prior to surgery. Samples from vitreous cavity were collected at the beginning and end of vitrectomy. All conjunctival specimens were cultured for different species and quantified using colony forming units (CFU).

**Results:**

There was a significant 82% reduction in the total number of CFUs for all bacteria in all eyes (*P* < 0.0001), and 90% reduction for coagulase negative staphylococci (CoNS) alone (*P* = 0.0002). The number of eyes with positive bacterial growth in conjunctival samples decreased from 33 to 18 after irrigation with CHX (*P* = 0.0023). The most common bacteria prior to surgery were CoNS (70%), *Propionibacterium acnes* (55%) and Corynebacterium species (36%). No case of post-vitrectomy endophthalmitis was reported during mean follow-up time, which was 4.6 ± 2.3 (range; 1.5 to 9) months.

**Conclusions:**

Patients undergoing PPV harbored bacteria in conjunctiva capable of causing post-vitrectomy endophthalmitis. Preoperative preparation with CHX significantly reduced the bacterial load in the conjunctival samples subsequently leading to very low inoculation rates in recovered vitreous samples. Thus, CHX used as a single disinfectant agent might be an effective preoperative procedure for eye surgery in Sweden. This is a relatively small study but the results could be a reference for other intraocular surgeries.

## Background

Postoperative endophthalmitis is a serious complication in intraocular surgery. Several different bacterial species are able to cause endophthalmitis after surgical procedures [1, 2], and sometimes with serious consequences and poor final visual outcomes [2, 3]. With the introduction of transconjunctival small-gauge vitrectomy (TSGV) there were reports of increased incidence of acute postoperative endophthalmitis [1, 3]. The commonly used preoperative disinfection of eyes in countries besides Sweden is polyvinylpyrolidone iodine (povidone iodine (PI)), which has been used for decades, and has been proven effective compared to other prophylactic measures by reducing bacteria (91%) on the ocular surface [4–7] . However, in Swedish health care, chlorhexidine (CHX) is the sole recommended antiseptic agent that is always used for preoperative washing preceding intraocular surgery and CHX was established as antimicrobial agent in 1954 [8]. It has a broad-spectrum bactericidal effect when used at different concentrations from 0.02 to 4% [9–11]. External use on skin and eyelashes includes chlorhexidine alcohol 0.5% and for preoperative irrigation of the ocular surface we use chlorhexidine solution 0.05% [12, 13]. Safety of CHX solutions for ophthalmic use was proved in experimental settings [14], and has been considered equally effective to PI in an outpatient study with 87% versus 91% reduction of bacteria [7]. Recently the safety and adequacy of aqueous chlorhexidine was demonstrated in a multicentre study from Australia where only 3 cases of endophthalmitis were identified in a total of 40,535 intravitreal injection (0.0074%, 1/13512) [15]. The ocular surface is colonized by bacteria and most post-surgical infections are probably originating from these bacteria. It has previously been suggested that an increased risk of intra-vitreous inoculation of pathogenic bacteria is due to transconjunctival approach of TSGV [16]. The trocar-valved cannula system will cut directly through the conjunctiva and into vitreous space and thereby potentially inoculate the vitreous with bacteria. Similar way of microorganisms entering the vitreous cavity was supported by the isolation of bacteria from used intravitreal injection needles [17]. Using molecular analysis of bacterial isolates, other authors demonstrated pathogenic bacteria isolated from vitreous cavity in patients treated from post-surgical endophthalmitis to be closely related to the bacteria isolated from the patients external ocular surface and nose [18]. The preoperative sterilization of the ocular surface should therefore probably be the primary objective to reduce bacteria of the ocular surface, and consequently the risk for endophthalmitis. We designed a prospective outpatient cohort study to assess the bacterial load in the conjunctival sac pre- and post-washing with CHX. We also examined the prevalence of introduced conjunctival flora into the vitreous cavity.

## Methods

### Patients

A total of 40 eyes of 39 consecutive elective patients who underwent TGSV with 23-gauge trocar (valved cannula) system at University Hospital in Örebro (UHÖ), Sweden, between years 2013 to 2014 were studied. A majority, 82% (32/39), of these outpatients was referred externally from other hospitals, and the follow-up was done at origin, but any post-operative endophthalmitis was treated surgically at the department of ophthalmology, UHÖ. Only patients with macular disease such as epiretinal membrane (ERM), macular hole (MH) and vitreomacular traction syndrome (VMT) were included in study. Exclusion criteria were; a history of vitrectomy or any other form of eye surgery during the last 2 months prior to surgery, any form of eye infection for the last 2 months preoperatively, any current systemic or local antibiotics therapy at the time of surgery, participating in any other study using eye medications, and patients with pathological myopic eye (≥ 6 diopter).

### Pre and postoperative procedures

A pre-irrigation sample (Start) was taken using a sterile flocked nylon swab (ESwab Copan liquid-based sampling system; Copan Diagnostics Inc., CA, USA) rolled back and forward once in the inferior conjunctival fornix. The eyelids were then gently retracted and the ocular surface was washed using 30 mL 0.05% chlorhexidine solution (Fresenius Kabi Ab; Uppsala, Sweden). After pausing for 8 ± 1 min, allowing CHX to drain from the conjunctival sac, the second sample (CHX-treated) was taken using the same procedure as the first sampling.

The eyelids and surrounding skin were cleansed with surgical compresses soaked in 0.5% chlorhexidine alcohol (Fresenius Kabi Ab), followed by sterile draping of lids and eyelashes according to a standard procedure. A single drop of topical antibiotic (chloramphenicol 0.5%; Trimb Healthcare AB, Stockholm, Sweden) was administered on the ocular surface once prior to surgery. All procedures were performed by three experienced surgical consultants.

The first vitreous specimen (V1) was collected at the beginning of vitrectomy, by using a sterile 2.0 mL syringe connected to the aspiration line of a high speed vitreous cutter. A sample of 0.5 to 0.7 mL of vitreous fluid was extracted from the vitreous cavity through controlled manual aspiration. The second sample (V2) (1.0 mL) was manually collected from the sterile infusion fluid (see below) suspension in the eye at the end of vitrectomy through the vitrector aspiration line before removal of the cannulas. No antibiotic was used in the sterile infusion fluid (Balanced Salt Solution (BSS), BSS PLUS; Alcon Laboratories Inc., Forth Worth, USA) during vitrectomy. The vitreous cavity was left with BSS at the end of surgery in 11 cases (27%), whereas gas or air tamponade was used in 29 cases (72%). The integrity of the scleral openings was tested and in 16 procedures (40%) of eyes sclerotomies needed to be sutured at the end of vitrectomy. At the end of the procedure a sub-conjunctival injection of Betapred 0.5 mL (betamethasone 4 mg/mL; Alfasigma S.P.A., Milano, Italy) and Zinacef 0.5 mL (cefuroxime 50 mg/mL; GlaxoSmithKline Ab, Solna, Sweden) were given in the inferior fornix. Postoperative treatment included topical drops of Isopto-Maxidex 1 mg/ml (dexamethasone; Novartis, Switzerland), 3 to 4 times in 4 to 6 weeks, and Cyclogyl 1% (cyclopentolate; Novartis) eye drops, 2 times in 2 weeks. The patients were not routinely treated with any antibiotics postoperatively. All procedures were performed by three experienced surgical consultants.

### Bacterial cultures

Conjunctival samples (50 μL) from the ESwab Copan medium (1 mL) were cultured on four differential agar plates (Enriched Chocolate Agar, Colombia Blood Agar, Fastidious Anaerobe Agar and Sabouraud Dextrose Agar). They were incubated aerobically or anaerobically for 7 days. One drop of vitreous aspirate (~30 μL) was inoculated to each of two selective agar plates (Enriched Chocolate Agar and Fastidious Anaerobe Agar) that were incubated as above. The remaining vitreous sample was distributed to supplemented aerobic and anaerobic culture bottles and incubated for 10 days in a Bactec FX-instrument (BD). Terminal subcultures were performed on all negative bottles. Conjunctival isolates were counted quantitatively in colony-forming units (CFU).

### Statistics

McNemar’s test for case-control comparisons of paired samples (two-tailed) were used for comparing culture result (±), pre- and post CHX-treatment. Wilcoxon Signed-Rank test for paired samples (two-sided) was used to evaluate the significance of the reduction in number of bacterial isolates and bacterial load (CFU). *P* values less than 0.05 were considered to be statistically significant. Values are expressed as mean ± standard deviation (SD). The calculations were performed using Graphpad Software programs (http://graphpad.com/quickcalcs/) (GraphPad Software, Inc. 2017), and IBM SPSS Statistics 22.0 (Statistical Packages for the Social Science, Chicago, IL, USA).

## Results

Demographic data of the eyes and culture results for all pre-irrigation (Start) and CHX-treated samples are summarized in Table 1. The mean patient age was 71.7 ± 5.6 (range; 63 to 85) years. No case of endophthalmitis occurred during the follow up time at the University hospital in Örebro or at referring centres. The mean follow-up time was 4.6 ± 2.3 (range; 1.5 to 9) months.

**Table 1:** Patient characteristics and bacterial isolates from conjunctival Samples, pre- and post-treatment with chlorhexidine (CHX)

Eye	Gender	Diagnosis	Bacterial isolates		Total CFU/mL	
			Start	CHX	Start	CHX
1	F	ERM	2+	2+	60	380
2	F	MH	3+	–	260	–
3	M	MH	1+	1+	360	240
4	F	MH	3+	–	500	–
5	F	MH	–	–	–	–
6	M	MH	3+	3+	4180	480
7	F	MH	1+	–	40	–
8	M	ERM	1+	–	40	–
9	M	ERM	1+	–	60	–
10	F	ERM	–	–	–	–
11	M	MH	–	–	–	–
12	F	MH	2+	1+	2040	60
13	F	ERM	3+	–	300	–
14	F	MH	–	–	–	–
15	F	ERM	1+	–	20	–
16	F	ERM	2+	3+	60	140
17	F	MH	2+	1+	60	20
18	F	ERM	2+	1+	160	40
19	M	MH	2+	1+	660	280
20	F	ERM	1+	–	60	–
21	M	ERM	3+	1+	620	40
22	M	ERM	2+	–	40	–
23	F	MH	–	1+	–	40
24	F	MH	1+	1+	140	20
25	F	ERM	1+	–	40	–
26	M	VMT	3+	–	540	–
27	M	ERM	3+	–	340	–
28	F	MH	–	2+	–	40
29	F	MH	2+	2+	2400	240
30	M	ERM	2+	–	1040	–
31	M	ERM	2+	1+	420	140
32	M	ERM	–	1+	–	120
33	F	ERM	2+	–	100	–
34	M	ERM	2+	1+	220	40
35	M	ERM	1+	–	60	–
36	M	ERM	3+	–	940	–
37	F	MH	1+	2+	1000	880
38	F	ERM	1+	–	1000	–
39	F	MH	1+	1+	200	40
40	F	ERM	1+	–	20	–
All culture positive eyes			33	18		
Reduction				45%		
*P* value				0.0023^a^		
All isolates/All CFU			61	26	17,980 CFU	3240 CFU
Reduction				57%		82%
*P* value				0.0004^b^		<0.0001^b^

Culture positive (+) isolates/sample: 1, 2 or 3. Culture negative (−). ^a^ McNemar’s test for case-control of paired samples (culture ±) two-tailed. ^b^ Wilcoxon Signed-Rank test for paired samples, two tailed

There was a significant 82% reduction in the total CFU in the eyes in almost all conjunctival samples, post CHX-treatment (Table 1).

Prior to CHX washing 33 of the eyes were culture positive including 61 isolates of 11 different groups of bacteria, and 1 to 3 different isolates/eye (Table 1).

After the preoperative washing with CHX the numbers of culture positive eyes were significantly reduced to 18 (*P* = 0.0023) and the proportion of culture positive eyes for the individual bacterial isolate was reduced. Bacteria were found in the vitreous samples of two patients. A CoNS was isolated after enrichment from vitreous sample (V1) in a healthy patient (eye no. 23, Table 1), and *P. acnes* was isolated after enrichment from second vitreous culture (V2) (eye no. 31, Table 1). Neither of these two patients had the isolated bacteria from the vitreous that matched the bacterial species that were cultured from conjunctiva.

Indications for surgery with different macular diseases are demonstrated in Table 1. One patient had diabetes mellitus (eye no.6) and 2 patients had psoriasis (eye no.17 and 28). The study included 22 phakic and 18 pseudophakic eyes. Intraoperative complications included 2 eyes with retinal tears. In only 16 eyes all 3 sclerotomies were sutured at the end of procedures. In 30 eyes were vitreous cavity left with air or gas at the conclusion of vitrectomy, while 10 eyes were left fluid filled. None of the patients experienced hypotony (defined as IOP ≤ 7 mmHg) on the first postoperative day.

The three most common bacterial isolates were reduced in both CFU, and the number of culture positive eyes (Table 2). The bacterial load for each isolate in the conjunctival sample varied between 20 CFUs to > 1000 CFUs/mL (Table 2).

**Table 2:** The bacterial load in eyes carrying the most commonly found isolates, pre- and post-chlorhexidine (CHX)

Bacteria	CoNS		*Propionibacterium acnes*		Corynebacterium species	
CFU/ mL	Start	CHX	Start	CHX	Start	CHX
20–40	11	8	6	3	2	0
41–200	10	1	5	2	4	1
201–1000	1	0	4	5	5	0
> 1000	1	0	2	0	1	0
All eyes	23	9	17	10	12	1
Reduction		61%		41%		92%
*P* value		0.0037^a^		0.0961^a^		0.0026^a^
Total CFU/mL	2900	300	9180	2440	4100	60
Reduction		90%		73%		99%
*P* value		0.0002^b^		0.0143^b^		0.0022^b^

^a^McNemar’s test for a case control of paired samples, two-tailed. ^b^Wilcoxon Signed-Rank test for paired samples, two tailed

The less common bacterial isolates were: *Staphylococcus aureus*, Beta- haemolytic Streptococcus grp.G, *Enterococcus faecalis*, Non-fermentative gram negative rod, Alfa- Streptococci, Anaerobic gram positive cocci, *Staphylococcus lugdunesis*, Micrococcus species. These were found in 1 to 3 eyes before and 0 to 2 eyes after CHX.

## Discussion

Preoperative preparation of the eye using CHX solution seems to be an efficient strategy for clearing ocular surface of bacteria and preventing intraocular infection. The rate of positive cultures from conjunctival samples decreased significantly in the total bacterial load (CFU), number of isolates and culture positive eyes, after washing with CHX 0.05%. The risk for perioperative inoculation of bacteria in the vitreous cavity was considered low during 23-gauge PPV as no case of endophthalmitis was observed in this study using this Swedish standard preoperative procedure. The number of positive conjunctival culture prior to CHX was comparable to previous reports (range; 67 to 80.2%) [12, 19], and as well as that CoNS as the most common culture positive result from an eye. The same bacterial flora was isolated from samples from vitreous cavity in patients treated for endophthalmitis [18]. Culture positivity in conjunctival samples from treated eyes was reduced by 45% after CHX irrigation*.* Also, the number of different bacterial isolates in the conjunctiva was reduced from 61 isolates to 26 isolates. Furthermore, the total number of detectable bacteria decreased by 82%, after rinsing of eye with CHX and even greater efficacy was observed for CoNS (90%). These major reductions in bacterial load, number of different bacterial genus/species and culture positive eyes were most likely the result of effective action of CHX solution and support its use as an antiseptic agent in Swedish eye surgery procedures. However, the complete surgical procedure used may also have influenced the outcome of results. Our findings are also comparable to results from other studies on disinfection methods in eye surgery used in other parts of the world. The use of povidone-iodine (5%), which reported a 91% decrease of number of colonies and 50 to 61% reduction of number of species after sterilization of ocular surface [4, 5] showed similar results as well as one CHX/PI comparative outpatient study [7]. Apart from commonly isolated bacteria like CoNS, Corynebacterium species and *P. acnes* other kind of bacteria were sparsely found only in the conjunctiva of a few eyes (Table 2). Among these, *S. aureus* and *E. faecalis* are important microorganisms causing post-surgical endophthalmitis with very poor visual outcomes despite emergency treatment [20].

In this study CoNS and *P. acnes* were isolated from vitreous samples (V1) respective (V2) (Table 1), both only positive after enrichment broth incubation. The source is therefore minute and of unclear significance. The risk for inoculation of bacteria into vitreous cavity in our series of 23-gauge TSGV (post–irrigation with CHX) seems low in comparison to 18% (18/98) using intravitreal injection [17]. Our bacterial isolation rate (2.5%) from the vitreous (V1) at initiation of 23-gauge vitrectomy was significantly lower than the rate of 22.5% (9/40 eyes) reported by Tominaga et al. during 25-gauge PPV [16]. However, at the completion of vitrectomy both studies reported comparable culture positivity (0 to 2.5%) from vitreous cavity (V2). Any vitreous contamination by low number bacteria of for example CoNS and *P. acnes* is probably removed during the vitrectomy extraction or secondary by the immune defense system of the patient preventing an intraocular infection. A positive bacterial culture from vitreous space at the end of vitrectomy does not necessarily lead to postoperative endophthalmitis [21]. At the Department of Ophthalmology, Örebro University Hospital, Örebro, Sweden, we experienced an unexpected increase of incidence of endophthalmitis during 2007 to 2012, using small 23-gauge vitrectomy (0.381%; 11/2885; 1 case per 262 patients, unpublished data N. J. Gili et al.). This was nearly 13 times higher compared to similar publications of Oshima et al. (0.030%; 2/6600) and Wu et al. (0.028%; 1/3615) [22, 23]. The endophthalmitis was caused by similar bacteria isolated in this current study. The present study demonstrates the clearance of colonizing bacteria in the conjunctiva, especially of CoNS, after washing with CHX. However, the individual patient samples were not always reduced after CHX. This could in at least one case (eye no. 1, Table 1) be the result of differences in sampling efficiency. The used culture methods and a limited detection level of 20 CFU could also influence the results (Table 1). No case of endophthalmitis was recorded during this study and follow-up. We might need to look at other causes and risk factors not presented in this study to find out the reasons for upsurge of endophthalmitis in 2007 to 2012. This study has some weaknesses. A relatively small number of patients were included. The surgical procedures and samplings were performed by three different surgeons in our department. There was also a risk for false negative result when analysing vitreous culture, due to difficulty in culturing low number of bacteria from small volume of vitreous sample.

## Conclusions

Current preoperative procedures used in Sweden include irrigation with chlorhexidine solution (CHX) 0.05% only and no iodine solutions. One drop of chloramphenicol was administered prior to surgery. We found that patients undergoing PPV harbored bacteria in conjunctiva capable of causing post-vitrectomy endophthalmitis. Preoperative preparation with CHX significantly reduced the bacterial load in the conjunctival samples subsequently leading to very low inoculation rates in recovered vitreous samples. Thus, CHX used as a single disinfectant agent may be an effective preoperative procedure for eye surgery in Sweden. This is a relatively small study but the results could be a reference for other intraocular surgeries. Further studies could give more information on the risks for endophthalmitis when using CHX only.

## Abbreviations

CFU: Colony-forming units
CHX: chlorhexidine
CHX-treated: Post-irrigation sample
CoNS: Coagulase-Negative Staphylococci
ERM: Epiretinal membrane
F: Female
M: Male
MH: Macular hole
PI: povidone iodine
PPV: Pars plana vitrectomy
SD: Standard deviation
Start: Pre-irrigation sample
TSGV: Transconjunctival small-gauge vitrectomy
UHÖ: University Hospital in Örebro
V1/V2: Vitreous specimens
VMT: Vitreomacular traction syndrome
